# A checkpoints capturing timing-robust Boolean model of the budding yeast cell cycle regulatory network

**DOI:** 10.1186/1752-0509-6-129

**Published:** 2012-09-28

**Authors:** Changki Hong, Minho Lee, Dongsup Kim, Dongsan Kim, Kwang-Hyun Cho, Insik Shin

**Affiliations:** 1 Department of Computer Science, KAIST, Daejeon, Korea; 2 Department of Bio and Brain Engineering, KAIST, Daejeon, Korea

**Keywords:** Timing robustness, Yeast cell cycle regulatory network, Model checking, Asynchronous Boolean networks

## Abstract

**Background:**

Cell cycle process of budding yeast (*Saccharomyces cerevisiae*) consists of four phases: G1, S, G2 and M. Initiated by stimulation of the G1 phase, cell cycle returns to the G1 stationary phase through a sequence of the S, G2 and M phases. During the cell cycle, a cell verifies whether necessary conditions are satisfied at the end of each phase (i.e., checkpoint) since damages of any phase can cause severe cell cycle defect. The cell cycle can proceed to the next phase properly only if checkpoint conditions are met. Over the last decade, there have been several studies to construct Boolean models that capture checkpoint conditions. However, they mostly focused on robustness to network perturbations, and the timing robustness has not been much addressed. Only recently, some studies suggested extension of such models towards timing-robust models, but they have not considered checkpoint conditions.

**Results:**

To construct a timing-robust Boolean model that preserves checkpoint conditions of the budding yeast cell cycle, we used a model verification technique, ‘model checking’. By utilizing automatic and exhaustive verification of model checking, we found that previous models cannot properly capture essential checkpoint conditions in the presence of timing variations. In particular, such models violate the M phase checkpoint condition so that it allows a division of a budding yeast cell into two before the completion of its full DNA replication and synthesis. In this paper, we present a timing-robust model that preserves all the essential checkpoint conditions properly against timing variations. Our simulation results show that the proposed timing-robust model is more robust even against network perturbations and can better represent the nature of cell cycle than previous models.

**Conclusions:**

To our knowledge this is the first work that rigorously examined the timing robustness of the cell cycle process of budding yeast with respect to checkpoint conditions using Boolean models. The proposed timing-robust model is the complete state-of-the-art model that guarantees no violation in terms of checkpoints known to date.

## Background

A cell must undergo the process of duplicating all its components and separating them, more or less evenly, to two daughter cells such that each daughter has the information and dynamics necessary to repeat the process. Such cell cycle dynamics are known in more detail for the budding yeast, *Saccharomyces cerevisiae*, compared to other eukaryotic organism [1,2]. The cell cycle process of budding yeast consists of four phases: G1, S, G2, and M. Initiated by stimulation of the G1 stationary phase, the cell cycle sequence proceeds (i.e., G1→S→G2→M) and finally returns to the G1 stationary phase. It is important to reach the final phase after completing each phase properly since any mistakes can cause significant defect to the cell cycle process. Hence, a cell verifies whether essential conditions are satisfied at checkpoints in G1, S, G2 and M phases, respectively. Before entering S phase, the cell must be large enough and have undamaged DNA (G1 phase checkpoint). Before entering M phase, DNA synthesis should be completed (S and G2 phase checkpoint). In M phase, the chromosomes need to be properly aligned and the spindles need to be oriented towards the daughter cell (M-metaphase checkpoint), and the cell should be correctly divided into two, before the end of mitosis (M-telophase checkpoint). The cell cycle can proceed to the next phase properly only if the above checkpoint conditions are satisfied.

Among several approaches [3-9] to capture the cell cycle process of budding yeast, one promising approach is to use Boolean network modeling, which is a widely used modeling framework in systems biology [10-18]. Claiming that Boolean models are useful for representing the cell cycle regulatory networks since much of the biology seems to be reflected in the on/off characteristics of the network components, the first Boolean model for the cell cycle regulatory network of budding yeast was introduced by Li, *et al.*[19]. Even with such a simple representation, they found that there exists a prominent dynamic gene expression trajectory satisfying the checkpoint conditions, and then it leads back to the G1 stationary state. It was also observed that essential conditions are robustly preserved at checkpoints under network perturbations. However, since the model employed synchronous update rules to represent state transitions such that all the nodes update their states synchronously at the same time, it lacks the timing robustness analysis of essential properties.

Timing robustness is the ability of a model to maintain its function in the presence of timing perturbations. Among a few ways to introduce various timing variations, Boolean modeling often uses an asynchronous updating of models. Unlike the synchronous update rule, the asynchronous update rule allows a maximum of one variable to be updated at each time instant, and if multiple variables are enabled to change, one of them is chosen arbitrarily. In this way, variations in reaction rates can be represented depending on the order in which the nodes update their values (i.e., some of the nodes update their values immediately while other nodes take longer). Here it is important to note that this way can generate a large number of distinct state transition trajectories, possibly as many as the number of all different order combinations. And such all different trajectories reflect every possible timing variation under dynamically changing environments. However, such a large number of trajectories to explore make it difficult to perform timing robustness analysis through biological simulation, since such simulation generally involves randomness for trajectory selection.

The main goal of this study is to construct a timing-robust Boolean model that properly preserves checkpoint conditions of the budding yeast cell cycle even in the presence of timing variations. Towards this goal, we used a model verification technique, ‘model checking’ [20], to cope with difficulties of timing robust analysis in conventional simulation based approach. Model checking is a method for formal verification of finite-state systems. A model checker, a software tool of model checking, explores all possible state transitions (i.e., all possible variations in reaction time) of a given model under fully asynchronous update rules. This way, we can then check exhaustively whether essential system properties always hold in the model or not. If the model contains a wrong state transition (i.e., hazard) that leads to a violation of system properties, a model checker automatically detects the hazard and produces a counter-example that can be used to pinpoint the source of the hazard.

In order to utilize model checkers, it needs to specify essential system properties in a form that model checkers can recognize, which is temporal logic [21]. Hence, based on the extensive literature studies [1,19,22-29], this study translates the critical checkpoint conditions of the budding yeast cell cycle into temporal logic formulas. It is worthy to note that such conditions should be properly preserved in order to proceed the cell cycle in the presence of timing variation. For example, an earlier wok by Mangla, *et al.*[30] aimed to extend the Li, *et al.*’s model towards a timing-robust model. Though this earlier work was able to construct a model that preserves the G1 stationary state in a timing-robust manner, it fails to capture essential checkpoint conditions completely against variations in reaction rates. It is also turned out that the checkpoint conditions violated in the Mangla, *et al.*’s model cannot be satisfied in the Li, *et al.*’s model as well under timing variations. We observed that such inadequate sequences of state transitions can cause a significant failure of the budding yeast cell cycle by violating the M phase checkpoint conditions. In particular, the both models allow a division of a budding yeast cell into two before the completion of its full DNA replication and synthesis. They have the gene expression trajectory that enables the gene Cdc20 to be activated before the activation of the gene Clb2, and such out-of-order sequential dynamics lead a transit to the M phase without completing the G2 phase.

In this study, we present a timing-robust model that properly preserves all the essential checkpoint conditions against timing variations. The proposed model is the complete state-of-the-art model that guarantees no hazard in terms of checkpoints known to date. As a result, our model naturally eliminates the hazards contained in the previous models (i.e., Li, *et al.*’s and Mangla, *et al.*’s model). Our simulation results show that the proposed timing-robust model is more robust even against network perturbations and can better represent the nature of cell cycle than previous models. The key to the success of the cell cycle process is to completely capture the checkpoint conditions phase-by-phase even in the presence of variations in reaction rates. To our knowledge this is the first work that rigorously examined the timing robustness of the cell cycle process of budding yeast with respect to checkpoint conditions using Boolean models.

## Results and Discussion

In this study, model checkers, software tools of model checking, are used to examine whether or not a specified logical property holds on every possible state of a Boolean model. The inputs to the model checker consist of a Boolean model, described as a set of variables and rules that update their values, an initial state, and a logical property to check. In this study, previously published Boolean models of the budding yeast cell cycle (i.e., the Li, *et al.*’s model and Mangla, *et al.*’s model) and the stimulated G1 state are used as input Boolean models and initial state to the model checker, respectively.

We derived the logical properties to check from essential checkpoint conditions of the budding yeast cell cycle. Based on the comprehensive literature studies [1,19,22-29], we found that the key regulators of the S, G2 and M phase checkpoints, Clb2 and Cdc20, are lethal genes. The activation of Clb2 initiates the M phase, and the activation and deactivation of Cdc20 trigger the metaphase to anaphase transition and the exit from mitosis, respectively. Since regulations of Clb2 and Cdc20 are closely related to the checkpoints, any damages of these genes can cause a fatal defect of the cell cycle process. Each checkpoint condition is translated into a group of specific sequences of state transitions that can be derived by ordering pairs of state transition among Clb2, Cdc20 and their interacting genes. For example, the M-metaphase checkpoint conditions can be rendered into two essential sequences of state transitions: Clb2 activation should precede Cdc20 activation; Mcm1 activation should precede Cdc20 activation. All essential properties derived from up-to-date checkpoint conditions are described in Additional file 1 with supporting evidences. Note that no property is derived from the G1 phase checkpoint because Boolean models based on the Li, *et al.*’s study do not completely include genes related to the checkpoint. Any state transitions violating such essential sequences of state transitions are called hazards. In this study, we used the NuSMV model checker [31] to construct a timing-robust model that properly preserves up-to-date essential checkpoint conditions of the budding yeast cell cycle. With such logical properties as inputs, the model checker detected two hazards in the previously published Boolean models (i.e., Li, *et al.*’s model and Mangla, *et al.*’s model) after conducting automatic and exhaustive state space search based on the fully asynchronous update rule. These hazards violate the M-metaphase (property 4, see Additional file 1) and M-telophase checkpoint (property 5-7, see Additional file 1), respectively.

### Timing robustness of the budding yeast cell cycle

**Figure 1:**
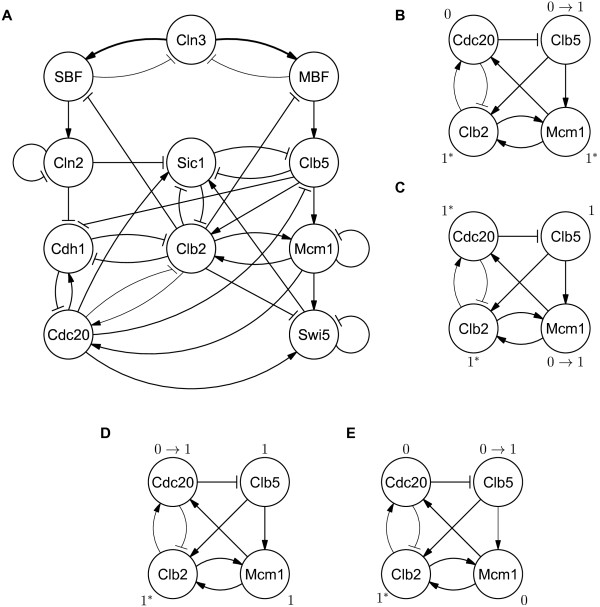
**Budding yeast model.** Nodes in the graph represent molecules. Lines with arrowhead represent the activation, and lines with flat ends indicate the inhibition. Thin arrows represent a low weight, a weight of 1/3, normal arrows indicate a medium weight, a weight of 1, and thick arrows represent a high weight, a weight of 3. (**A**) The model from Mangla, *et al.*[30]. (**B**) A subset of the model that highlights the first hazard. Nodes with values marked with * are enabled to change to the values. If Clb5 transitions to 1, both Clb2 and Mcm1 are eligible to change their values to 1 at the next time step. (**C**) If Mcm1 activates first, Cdc20 is enable to update its value as well as Clb2. (**D**) If Cdc20 activates before Clb2 transitions to 1, the first hazard occurs. (**E**) The hazard can be eliminated by replacing the weight of the reaction between Clb5 and Mcm1 to the one with the lower weight.

The first hazard in the Mangla, *et al.*’s model is shown in Figure 1. The hazard can lead the model to a biologically undesirable situation, in which it enters the M phase even though the DNA synthesis process is not complete. It violates the property 4 of the M-metaphase checkpoint conditions (see Additional file 1). Such inadequate state transition can occur when Clb5 gets activated in the model. After Clb5 transitions to 1, both Clb2 and Mcm1 are enabled to change from 0 to 1 (Figure 1B). There are two cases, depending on which of Clb2 and Mcm1 changes first. If Clb2 changes first, the cell cycle normally proceeds to the M phase through the G2 phase, guaranteeing the completion of DNA synthesis [23]. However, if Mcm1 changes first, Cdc20 is enabled to change from 0 to 1 (Figure 1C). As shown in Figure 1D, the first hazard is detected when Cdc20 transitions to 1 before the activation of Clb2, which means that the division of a cell into two can begin before the completion of DNA replication. Note that the activation of Clb2 is required for the proper cell progression to the M phase and that Cdc20 becomes active after Clb2 phosphorylates APC core proteins (e.g., Cdc16, Cdc23, and Cdc27) [23]. There can be a further timing gap between the activation of Clb2 and Cdc20 in reality because, even if the phosphorylated form of the APC is bound to Cdc20, Cdc20 becomes active only after the chromosomes properly align in the metaphase stage.

The Mangla, *et al.*’s model can be revised to eliminate the first hazard by adjusting the weight of an edge. When the weight of the edge from Clb5 to Mcm1 decreases to a low level, it ensures that when Clb5 is activated, only Clb2 is able to be subsequently activated, but Mcm1 is not (Figure 1E). Note that either of Clb2 and Mcm1 can be activated in any order in the Mangla, *et al.*’s model. Chen, *et al.*[22] supports this revision, showing that Clb5 makes a stronger reaction to Clb2 than to Mcm1. Other works [32,33] also support this revision, revealing that Clb2 and Mcm1 make positive feedbacks; Mcm1 is activated by the low level activation of Clb2, and then the activation of Mcm1 causes Clb2 to be activated at a higher level of concentration in return. Such a biological evidence was reflected in part to the Mangla, *et al.*’s model such that Clb2 has one of three possible values: 0, representing a negligible concentration of Clb2; 1, representing a low concentration; and 2, representing a high concentration. However, the Mangla, *et al.*’s model does not capture the positive feedback completely. In the model, Clb2 is able to be activated to at high concentration (i.e., the level of 2) without forming positive feedback. We revised the Mangla, *et al.*’s model by eliminating the first hazard, and the revised model is able to capture the proper dynamics due to the positive feedback between Clb2 and Mcm1.

**Figure 2:**
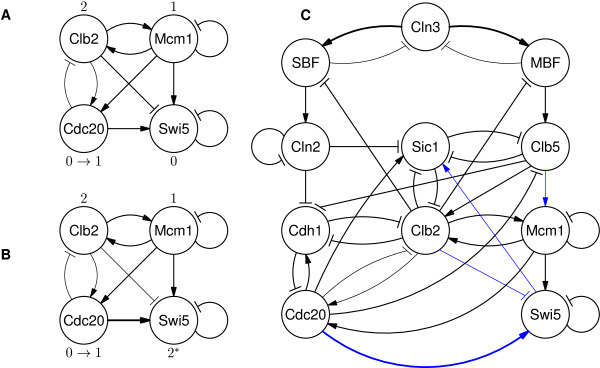
**Budding yeast model. **(**A**) A subset of the Mangla, *et al.*’s model that highlights the second hazard. Since Clb2 is activated to the highest level, the activation of Cdc20 cannot activate Swi5. (**B**) The hazard can be eliminated by replacing the weights of reactions, from Cdc20 and Clb2 to Swi5, to stronger and weaker levels, respectively. Swi5 is also extended to have one of three eligible values like Clb2. (**C**) The final timing-robust model for the budding yeast cell cycle. Lines with blue are modified from the Mangla, *et al.*’s model.

The second hazard violating the properties 5 to 7 of the M-telophase checkpoint conditions (see Additional file 1) occurs after Cdc20 transitions to 1 (Figure 2A). Since the activation of Swi5 is mostly dependent on the activation of Cdc20 [22], Swi5 should be activated when Cdc20 transitions to 1 regardless of the activation level of Clb2. In the Mangla, *et al.*’s model, however, Swi5 is able to change its state only when the activation level of Clb2 decreases to 1 from 2; if the concentration level of Clb2 is 2, Swi5 remains deactivated until the level of Clb2 becomes 1 even though Cdc20 is activated. Such inadequate state transition can cause the model to inevitably delay the exit from mitosis. The complete division of a budding yeast cell related gene, Sic1, is deferred to be activated since the activation of the transcription factor of Sic1 (i.e., Swi5) became delayed.

According to the study by Chen, *et al.*[22], the activation of Mcm1 causes Swi5 to be transcribed at a low level, and then Swi5 is activated at a higher level after Cdc20 is activated. To capture this understanding, we revise the model so that Swi5 also has one of three possible values: 0, 1, or 2 like Clb2. In addition, the model is revised such that the reactions from Cdc20 and Clb2 make stronger and weaker impacts on Swi5, respectively. This revision ensures that Swi5 is activated to 1 by its transcription factor, Mcm1, and then activated to 2 after Cdc20 is activated regardless of the activation level of Clb2, since reduced inhibition from Clb2 cannot dominate the level of transcription of Swi5 anymore (Figure 2B). In accordance with previous observation [22], the model is additionally revised to have a weaker reaction from Swi5 to Sic1. This revision guarantees the reaction from Swi5 to have impact on the activation of Sic1 only when the concentration of Swi5 increases to the highest level of 2.

Figure 2C represents the final hazard-free model for the budding yeast cell cycle. We found that the checkpoint conditions violated in the Mangla, *et al.*’s model cannot be satisfied in the Li, *et al.*’s model as well. To our knowledge, the proposed model is the first timing-robust model that properly captures up-to-date checkpoint conditions of the budding yeast cell cycle in the presence of variations in reaction rates.

### Temporal evolution of gene states for budding yeast cell cycle models

Mathematical modeling based on biochemical rate equations, provides a rigorous and reliable tool for unraveling the complexities of molecular regulatory networks. However, this approach is only suited for small and well-characterized systems with known kinetic parameters since there is a lack of detailed knowledge of quantitative reaction kinetics for most of the reactions in a cell [1,34]. Fortunately, the cell cycle regulatory system of budding yeast is most fully worked out, so its control system is revealed in exquisite details. The mathematical model of the budding yeast cell cycle has been published by Chen, *et al.*[22], and the model is widely used due to its acceptable accuracy in explaining a real cell [35,36].

To investigate how closely Boolean models of the budding yeast cell cycle represent nature, we compared the temporal evolutions of gene states in Boolean models to that in the mathematical model. Note that the asynchronous update rule allows a maximum of one variable to be updated at each time instant, and if multiple variables are enabled to change, one of them is chosen in an arbitrary fashion. Thus, too many different state transitions can exist under the asynchronous update rule. When applying the synchronous update rule to Boolean models for simple comparison, we observed that the temporal evolution of gene states in the proposed model maintains a similar structure to those in the other Boolean models overall. However, we also found that the extension from the Mangla, *et al.*’s model leads some genes in the proposed model to evolve analogously to the dynamics of the corresponding genes in the mathematical model in some period, and such genes are closely related to the hazards which are eliminated during the extension.

**Figure 3:**
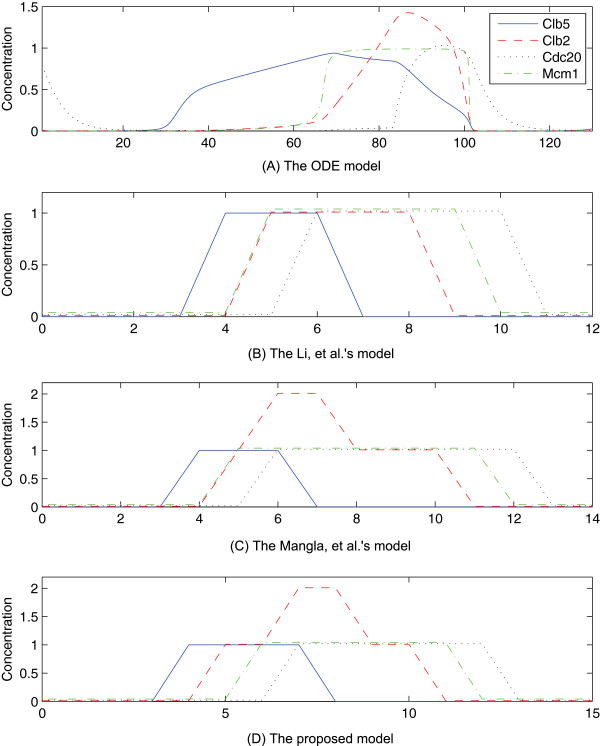
**Temporal evolution of variables related to the first hazard in the budding yeast cell cycle model.** X-axis and y-axis represent the time and the level of activation concentration in simulation, respectively. (**A**) The low level activation of Clb2 through the time of 30 to 45 causes the transcription of Mcm1. Finally, Clb2 is activated to a high level by Mcm1. (**B**)-(**C**) At the time of 5, both Clb2 and Mcm1 are activated and do not form a positive feedback. (**D**) Clb2 is first activated at the time of 4, and then Mcm1 is activated. After the transcription of Mcm1, Clb2 is activated to value of 2, a high activation level, at the time of 7.

As shown in Figure 3A, the mathematical model clearly shows a positive feedback between Clb2 and Mcm1: Clb2 is transcribed at a low concentration in the interval from 30 to 45, which is sufficient to activate Mcm1, and then Clb2 is transcribed at a higher level at the time of 50 via the activation of Mcm1. The temporal evolution of the proposed model also forms positive feedback as shown in Figure 3D, but the other two models cannot result in such a relationship since both Clb2 and Mcm1 are activated simultaneously at the time of 5. In the application of the asynchronous update rule, those previously published Boolean models (Figures 3B and 3C) can generate the hazard since Cdc20 can be activated before the transcription of Clb2 if Mcm1 is activated first under the condition that both Clb2 and Mcm1 are enabled to change.

**Figure 4:**
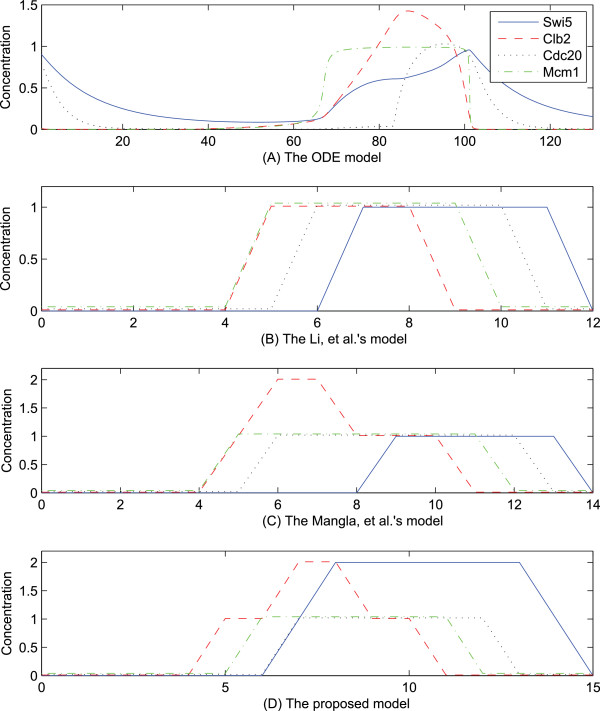
**Temporal evolution of variables related to the second hazard in the budding yeast cell cycle model.** X-axis and y-axis represent the time and the level of activation concentration in simulation, respectively. (**A**) Swi5 is activated to a low level by Mcm1, and then transcribed to a high level via the activation of Cdc20 at the time of 80. (**B**) Swi5 is inactive until Cdc20 is activated regardless of the activation of Mcm1. (**C**) Swi5 is deactivated until Cdc20 is activated regardless of the activation of Mcm1. (**D**) Swi5 is activated to a low level by the activation of Mcm1 at the time of 6, and then transcribed to a high level via the activation of Cdc20 at the time of 8.

Moreover, the temporal evolution of Swi5 in the proposed model is closer to the dynamics of the gene in reality (Figure 4D). Consistent with the mathematical model, Swi5 in the proposed model is first transcribed at a low concentration (i.e., a value of 1) at the time of 7 by the activation of Mcm1. It is then activated to a high concentration (i.e., a value of 2) at the next time step after Cdc20 is activated. However, in the other Boolean models, it is shown that Swi5 is still in the inactive state even after Mcm1 becomes activated. Specifically, Swi5 is deactivated until Clb2 is degraded to the low level of activation regardless of the activation of Cdc20 in the Mangla, *et al.*’s model.

From this simulation, it appears that the proposed model follows similar dynamics as those in the other Boolean models, even if the proposed model is extended from the others. In addition, in the proposed model, the temporal evolution of genes related to the extension shows better consistency with the one in the mathematical model of the budding yeast cell cycle. Therefore, the proposed model which is constructed by eliminating hazards in the previous models better reflects the nature of cell cycle than the previously published Boolean models.

### Relative durations of each cell cycle phase

Many efforts have been made to discover checkpoints and key transcription factors responsible for phase transitions in cell cycles [1,19,22-29]. Therefore, it is useful for cell cycle models to reflect properties on the duration of each phase in a cell cycle.

Recent studies have assumed that every edge in the yeast cell cycle regulatory network proceeds with the same speed since transcriptions normally happen on similar time scales [37,38]. Following this assumption, we compared the average length of state transitions in each phase in the models, referring to the relative duration of each phase in nature. The S phase begins when the state of the model is the same as the stationary G1 phase except that Cln3 is 1. Table 1 shows that in the Li, *et al.*’s model, the average length of the S phase is 9.30 and the average length of the G2/M is 7.56. In the Mangla, *et al.*’s model, the average length of the G2/M becomes longer, which is 10.89. These results are inconsistent with the experimental data, in which it was observed that the relative duration of the G2/M phase is twice as long as that of the S phase [39,40]. However, in the proposed model, the G2/M phase is almost twice as long on average than the S phase. In our understanding, this is because the S phase becomes shorter with our revision of the Mangla, *et al.*’s model with regard to positive feedback between Clb2 and Mcm1, and the G2/M phase becomes longer with our extension of Swi5’s activation level, which produced additional state transitions to reach the end of the M phase.

**Table 1:** Average length of state transitions for each phase and their variation

**Phase**	**The Li, *et al.*’s model**		**The Mangla, *et al.*’s model**		**The proposed model**	
	**Average**	**Variation**	**Average**	**Variation**	**Average**	**Variation**
S	9.30	1.55	9.30	1.55	7.47	0.41
G2/M	7.56	1.25	10.89	2.14	12.52	1.72

Consistent with the experimental data [39,40], the average length of the G2/M phase is about two times longer than the one of the S phase, in the proposed model.

### Attractor analysis

We used the proposed model to study the attractors of the network dynamics by starting from each of the 2^9^×3^2^=4,608 states in the 11-node proposed model with two 3-valued nodes. We found that all of the initial states eventually flow into one of the nine stationary states, also called attractors (Table 2). The basin size of an attractor is the number of initial states which reach the attractor after a finite number of time steps. As seen in Table 2, there is one big fixed point which attracts 4,323 or ≈ 94% protein states from 4,608 initial states. This is consistent with a previous study [19] which reveals that the model has one big attractor, and the dominant attractor is the biological G1 stationary state. Interestingly, the basin size of the big attractor in the proposed model is much larger than the one presented in the Li, *et al.*’s model (i.e., 1,764 or ≈ 86%, Table 3). It is also larger than the one in the Mangla, *et al.*’s model (i.e., 2769 or ≈ 90%, Table 4). It is obvious that the proposed model is more stable than the other models of budding yeast because the big attractor represents a cell’s stationary state, and the basin size is the largest in the proposed model. Under normal conditions, a cell will be sitting at the state that the biggest attractor represents, waiting for another round of divisions.

**Table 2:** Attractors and their basin sizes of the proposed model

**Basin size**	**Cln3**	**MBF**	**SBF**	**Cln2**	**Cdh1**	**Swi5**	**Cdc20**	**Clb5**	**Sic1**	**Clb2**	**Mcm1**
4323	0	0	0	0	1	0	0	0	1	0	0
87	0	1	1	1	0	2	1	0	1	0	0
68	0	0	1	1	0	0	0	0	0	0	0
46	0	1	0	0	1	0	0	0	1	0	0
44	0	0	0	0	0	0	0	0	0	0	0
16	0	0	1	1	0	2	1	0	1	0	0
12	0	0	0	0	1	0	0	0	0	0	0
10	0	0	0	0	0	0	0	0	1	0	0
2	0	1	0	0	0	0	0	0	1	0	0

Each attractor is represented in a row. The first column is the size of the basin of attraction for the attractor. The other 11 columns show the protein states of the attractor. In the proposed model, the biggest fixed point representing the stationary G1 phase attracts 4,323 or ≈ 94% from 4,608 initial states.

**Table 3:** **Attractors and their basin sizes of the Li, *et al.*’s model**

**Basin size**	**Cln3**	**MBF**	**SBF**	**Cln2**	**Cdh1**	**Swi5**	**Cdc20**	**Clb5**	**Sic1**	**Clb2**	**Mcm1**
1764	0	0	0	0	1	0	0	0	1	0	0
151	0	0	1	1	0	0	0	0	0	0	0
109	0	1	0	0	1	0	0	0	1	0	0
9	0	0	0	0	0	0	0	0	1	0	0
7	0	1	0	0	0	0	0	0	1	0	0
7	0	0	0	0	0	0	0	0	0	0	0
1	0	0	0	0	1	0	0	0	0	0	0

Each row and column represents the same as Table 2. In the Li, *et al.*’s model, the biggest fixed point representing the stationary G1 phase attracts 1,764 or ≈ 86% from 2,048 initial states.

**Table 4:** **Attractors and their basin sizes of the Mangla, *et al.*’s model**

**Basin size**	**Cln3**	**MBF**	**SBF**	**Cln2**	**Cdh1**	**Swi5**	**Cdc20**	**Clb5**	**Sic1**	**Clb2**	**Mcm1**
2769	0	0	0	0	1	0	0	0	1	0	0
159	0	1	1	1	0	1	1	0	1	0	0
52	0	0	1	1	0	0	0	0	0	0	0
44	0	1	0	0	1	0	0	0	1	0	0
18	0	0	1	1	0	1	1	0	1	0	0
17	0	0	0	0	0	0	0	0	1	0	0
7	0	0	0	0	0	0	0	0	0	0	0
5	0	1	0	0	0	0	0	0	1	0	0
1	0	0	0	0	1	0	0	0	0	0	0

Each row and column represents the same as Table 2. In the Mangla, *et al.*’s model, the biggest fixed point representing the stationary G1 phase attracts 2,769 or ≈ 90% from 3,072 initial states.

### Number of different state transitions and timing robustness

Table 5 shows the number of different state transitions for each cell cycle phase, and the major difference among the three models is found in the G2 phase. To investigate the relationships between the number of different state transitions and the timing robustness of the model, we introduced random mutations into the models. To perturb only an interested cell cycle phase, we applied mutations to edges related to the corresponding phase by deleting, adding, or re-weighting them. In this way, 200 mutant networks were generated, and then checked whether or not the dynamics of networks preserve the G1 stationary state as their global attractor under the fully asynchronous update rule using the NuSMV model checker. In the phase transition from the G2 phase to the M phase, the Mangla, *et al.*’s model was found to be vulnerable to mutations (Table 6), and the fraction of timing-robust mutants holding the cell cycle property was much smaller than in the proposed model. It was even smaller than those from the other phases in the same model. This is interpreted to indicate that incomplete construction of the positive feedback between Clb2 and Mcm1 in the Mangla, *et al.*’s model causes the G2 phase to be fragile to perturbations. In addition, the extension of the budding yeast cell cycle model by elimination of such hazards increases the number of viable state transitions to correctly arrive at the M phase from the G2 phase, which allows the proposed model to have about 10% greater timing robustness. The Li, *et al.*’s model includes hazards which drives the model to different attractors rather than the G1 stationary state. Thus, mutants of the model are also unlikely to hold the property, as shown in Table 6.

**Table 5:** Number of different state transitions for each phase

**Phase**	**Li, *et al.***	**Mangla, *et al.***	**Proposed**
	**Number of state transitions**	**Number of state transitions**	**Number of state transitions**
G1	51	51	51
S	6	6	1
G2	9	35	215
M	3	4	4

The major difference among the three models in terms of the number of different state transitions for each cell cycle phase is found in the G2 phase.

**Table 6:** Fraction of timing-robust mutants for each phase

**Phase**	**Mutation**	**Li, *et al.***	**Mangla, *et al.***	**Proposed**
	**distance**	**% of timing-robust**	**% of timing-robust**	**% of timing-robust**
	1	0.00	0.31	0.31
G1	2	0.00	0.22	0.22
	3	0.00	0.15	0.15
	1	0.00	0.26	0.22
S	2	0.00	0.12	0.20
	3	0.00	0.07	0.10
	1	0.00	0.17	0.27
G2	2	0.00	0.09	0.21
	3	0.00	0.08	0.12
	1	0.00	0.22	0.28
M	2	0.00	0.14	0.16
	3	0.00	0.13	0.13

The second column represents the number of mutations applied to a model. To perturb only an interested cell cycle phase, we applied mutations to edges related to the corresponding phase by deleting, adding, or re-weighting them. In this way, 200 mutant networks were generated, and then checked whether they preserve the desired attractor, the G1 stationary state under the asynchronous update rule using NuSMV. Columns from third to fifth represent the fraction of timing-robust mutants in each model.

We conjecture that the network robustness of a model is closely related to the number of different biological state transitions which satisfy properties of the model. This is because even if some state transitions are altered by mutations, the remaining transitions are likely to hold biological properties of the model, and our simulation results demonstrate this.

### Limitations

The proposed model is the timing-robust model that properly preserves all the essential checkpoint conditions against timing variations. Although the proposed model is the complete state-of-the-art model that guarantees no hazard in terms of checkpoints known to date, it still has a gap with quantitative models. Our model is not yet directly applicable to explaining and predicting the quantitative outcome of biological experiments of the budding yeast cell cycle. Quantitative models can potentially describe molecular interactions with high precision and in quantitative terms that correspond to realistic laboratory measurements. However, Boolean models can still be used by a subset of researchers because of easy understanding of the dynamics of the budding yeast cell cycle. We expect that the proposed model can be used to help them by providing a more stable and timing-robust Boolean model.

The main obstacle in application of model checking in practice is the state space explosion problem [20]. Since model checkers should examine all the possible model states, the number of states can grow exponentially in the number of program variables. When verifying a large-scale biological system, it is intractable to explore the entire state spaces because they exceed computational limits (i.e., time and memory). This problem is known as state space explosion. The last 30 years have seen various techniques for resolving the state space explosion problem [41]. In particular, several techniques have been introduced to decrease the number of states to be explored and the memory requirements needed for storing explored states, leading to substantial reduction in state space [42]. We further expect that biological experts can propose biology-specific insight and knowledge to state space reduction techniques, such as biological abstraction that allows a set of biologically equivalent states to be considered as a single symbolic state, resulting in significant state space reduction.

## Conclusions

Timing robustness analysis is one of the most important and challenging problems in systems biology [43-45]. It is critical for biological systems to maintain its essential dynamics robustly under various reaction delays in dynamically changing environments [46-49]. Pointing out that time delays are common and substantial in gene regulatory networks, Chen, *et al.*[50] proposed a method to design robust gene regulatory networks under biochemical timing variations and molecular noises. Lopez-Aviles, *et al.*[51] revealed that time delays have a significant influence on fundamental dynamics by showing that the unidirectionality of eukaryotic cell cycle transitions (i.e., G1→S→G2→M→G1) can hold only after a certain amount of time delays of cyclin degradation.

Boolean network modeling, which is now a widely used modeling framework in systems biology, requires timing robustness analysis since reaction kinetic parameters inevitably vary over a certain range. However, most researchers paid little attention to timing robust analysis so far and assumed that Boolean models are updated in a synchronous manner, neglecting timing variations [30]. Considering the importance of robustness of biological systems over timing variations, timing robustness analysis needs to be carefully considered in most Boolean network models.

A number of experimental studies on budding yeast, *Saccharomyces cerevisiae*, illuminated its cell cycle dynamics in greater detail than any other eukaryotic organisms [1,2]. Such rich experimental results have fostered a growing attention to modeling of the budding yeast cell cycle network as it is recognized as a good benchmark example for studying the fundamental design principles behind the well-orchestrated behavior [3-9]. In particular, Li, *et al.*[19] presented a yeast cell cycle network model that has brought a substantial influence on other studies on the yeast cell cycle. It provided a basis for constructing more robust models under stochastic environmental fluctuation [52-55]. Other studies extended the Li, *et al.*’s model by incorporating activation/deactivation delays with the auxiliary nodes [24,56].

Building upon the Li, *et al.*’s model, Mangla, *et al.*[30] was able to construct a model that preserves the G1 stationary state in a timing-robust manner, it fails to capture essential checkpoint conditions completely against variations in reaction rates. Hence, we proposed here a timing-robust Boolean model that properly preserves up-to-date checkpoint conditions of the budding yeast cell cycle. Our model provides a basis for other subsequent further studies on budding yeast cell cycle analysis with Boolean network models that would result in biologically more robust results.

Boolean modeling and analysis of complex biological networks aim to provide a system-level understanding on complex biological phenomena [10-18]. In practice, however, constructing Boolean network models from biological data typically requires a significant amount of manual efforts through repetitive modeling and checking processes; models are commonly revised iteratively until they conform with targeted essential behavior of biological systems [57-60]. Model checking could facilitate such repetitive designing processes by performing automatic checking of biologically plausible models and suggesting new testable predictions upon every model-based simulation failure [11,20,61-65]. For instance, Fisher, *et al.*[61] inferred new regulation of inductive and lateral signaling crosstalk of *C. elegans* according to the testable predictions suggested by a model checker and then confirmed the newly inferred regulation with biological experiments.

In systems biology, mathematical models are becoming too complicated to be validated by examining some essential dynamics in an ad hoc way. It becomes even more difficult to check manually whether a combination of dynamics (e.g., ordered dynamics) are met simultaneously in complex biological networks [66]. On the other hand, model checking can validate a set of essential functionalities and their combinations in an automatic manner. So, model checking can be beneficial to modeling and analysis of biological systems, particularly for developmental systems [67] and cell-fate decision systems [68] where it is crucial to maintain ordered dynamics over environmental variations. In this respect, our model checking-based approach can be useful and provide a systematic framework for robustness analysis.

## Methods

### NuSMV

Model checking [20] is a technique for the verification of correctness properties of finite-state systems. One benefit of this technique is that systems can be automatically verified by use of a tool, called a *model checker*. The system is translated into a model described in the input language of the model checker. The model checker explores every state space of the given model to check whether system properties hold in the model or not. If the model violates any of the system specifications, the model checker produces an execution trace (i.e., counter-example) showing why the specification turns out false in the model. Counter-examples usually serve as good hints when a model is revised.

We used a symbolic model checker, NuSMV [31], as in the study by Mangla, *et al.*[30]. NuSMV can easily switch its modes in exploring state spaces between synchronous and asynchronous manners by introducing a single control variable. NuSMV, therefore, is useful to test whether a budding yeast cell cycle model satisfies its own cell cycle properties both in synchronous and asynchronous manners. We chose a Boolean decision diagram (BDD)-based implementation for the exploration of state spaces among various implementation candidates. Traditional representation of Boolean functions generates redundant state spaces explicitly when exploring state spaces. On the other hand, the BDD data structure can keep much smaller state spaces in the exploration of state spaces than the traditional ones do. Thus, BDD is beneficial in dealing with the state space explosion problem, which is one of the main challenges of model checking techniques for the verification of large-scale systems.

A NuSMV model consists of one or more modules. Each module can declare variables and their update rules. Variables can be declared to have a range of discrete values. The rules specify how to initiate variables and update them at every time step from their current values. Update rules can be non-deterministic since they can result in different values of a variable under the same condition. NuSMV checks whether a given property holds over all different possibilities. In describing our budding yeast cell cycle model in the NuSMV input language, we assign a single variable to each node of the model and specify the update rules of every variable. By default, variables in the NuSMV model are updated in a synchronous manner. There is a global clock and all modules execute in parallel every time the global clock ticks. To apply the asynchronous update rule to the NuSMV model, we define an additional control variable. At each time step, the control variable indicates which variable to update in a non-deterministic manner such that only the update rules of the chosen variable can execute in the model. In addition, we add a *FAIRNESS* property to every variable to prevent the control variable from repeatedly choosing the same variables for update, which would cause other variables not to be chosen for a long time.

Programs in the language can be annotated by properties expressed in temporal logic, that is, computation tree logic (CTL) [21]. Besides the properties describing the sequences of transitions between states, some properties such as “*eventually*” or “*never*” can also be specified with special temporal operators in CTL. For example, cell cycle properties (e.g., a cell starts from the G1 phase, and *eventually* returns to the G1 stationary phase after a division; Cdc20 activation should *never* begin before Clb2 activation) can be encoded by CTL formulas.

### Boolean model construction

In this paper, we presented Boolean networks of the budding yeast cell cycle. Boolean models include nodes and edges for different components and interactions of the system, respectively. Each node in the Boolean model has one of two values: 1 for ON (active) and 0 for OFF (inactive). A state *S* of each node *i* at any time instant *t* (denoted as *S*_*i*_(*t*)) is determined according to a Boolean function (rule) and the states of its input nodes at the previous time instant *t*−1. Most Boolean functions are threshold based [18]. When the value of a node is updated, it is assigned one if the weighted sum of positive and negative inputs exceeds a pre-defined threshold for that node. For example, the Li, *et al.*’s model describes its Boolean rule as follows [19]:

$$S_{i}\left(t\right)=\left\{\begin{array}{cc} 1,\;\;\; & \underset{j}{\sum }w_{\mathrm{ij}}S_{j}(t-1)>\theta_{i}\\ 0,\;\;\; & \underset{j}{\sum }w_{\mathrm{ij}}S_{j}(t-1)<\theta_{i}\\ S_{i}(t-1),\; & \underset{j}{\sum }w_{\mathrm{ij}}S_{j}(t-1)=\theta_{i}, \end{array}\right)$$

where *w*_*ij*_represents the weight of an incoming edge to a node *i* from a node *j*, and the threshold (denoted by *θ*_*i*_) is set to zero.

A synchronous Boolean model is one of the simplest implementations for the application of such Boolean update rules to nodes. In such a model, a Boolean update rule is applied to all the nodes simultaneously at each time instant. Synchronous models are deterministic since nodes are assumed to work in the same time scale, resulting in convergence to the same state from the same initial condition after the same number of time steps. As the result of applying the Boolean function described above synchronously, the first synchronous Boolean model for the budding yeast cell cycle, the Li, *et al.*’s model, converged into seven attractors from 2^11^ initial states in the 11-node network model.

Although synchronous Boolean models have been widely used due to their simple nature and ease of implementation, they lack consideration of a variety of time scales in biological systems. To deal with this drawback, asynchronous models are suggested in which a maximum of one node is chosen to be updated at each time instant. Since it is usually unknown exactly how long specific biological processes take, most asynchronous algorithms are non-deterministic in a way that a single node is randomly chosen at each time unit. The Mangla, *et al.*’s model is constructed by the use of the NuSMV model checker such that it can preserve its desirable attractor, the G1 stationary state in every possible combination of node updating order. The model is designed to be independent of whatever time scales biological reactions are subject to; thus the model is considered timing-robust model. To construct a timing-robust budding yeast cell cycle model dealing with the hazards of the Li, *et al.*’s model, the Mangla, *et al.*’s model allows Clb2 to have one of three possible values (i.e., 0, 1 or 2) and edges assigned to one of three magnitudes of weights (i.e., $\left(\pm \frac{1}{3}\right)$, ±1 or ±3). The Boolean rule is also defined, extending that of the Li, *et al.*’s model, as follows:

$$S_{i}\left(t\right)=\left\{\begin{array}{l} S_{i}(t-1)-1,\;\underset{j}{\sum }w_{\mathrm{ij}}S_{j}(t-1)<\theta_{i,S_{i}(t-1)}\\ S_{i}(t-1)+1,\;\underset{j}{\sum }w_{\mathrm{ij}}S_{j}(t-1)\geq \theta_{i,S_{i}(t-1)+1}\\ \;S_{i}(t-1),\;\text{otherwise.} \end{array}\right)$$

In the model, the thresholds for the value of 1 (denoted by *θ*_*i*,1_) and 2 (denoted by *θ*_*i*,2_) for a node *i* are set to 0.5 and 1.5, respectively.

Basically, the model proposed in this study extended a set of available values and weights for each node and edge, respectively. The proposed model also follows the same Boolean function as the Mangla, *et al.*’s model follows, but it refined the Mangla, *et al.*’s model to hold essential checkpoint conditions as well. It finally became the timing-robust model that captures up-to-date checkpoint conditions of the budding yeast cell cycle.

## Competing interests

The authors declare that they have no competing interests.

## Author’s contributions

CH and IS jointly conceived the study, and wrote the manuscript. ML and DK (Dongsan Kim) interpreted simulation results and helped draft the manuscript. DK (Dongsup Kim) and KC critically reviewed the manuscript. All authors read and approved the final manuscript.

## Supplementary Material

Additional file 1 **Essential ordered properties derived from checkpoints.** The PDF file contains a list of all the essential ordered properties derived from the up-to-date checkpoint conditions. Click here for file
