# Supplementary material for: A checkpoints capturing timing-robust Boolean model of the budding yeast cell cycle regulatory network
Source: BMC Syst Biol. 2012 Sep 28;6:129. doi: 10.1186/1752-0509-6-129 (PMC3573974; doi:10.1186/1752-0509-6-129)
Supplement: Additional file 1 — Essential ordered properties derived from checkpoints. The PDF file contains a list of all the essential ordered properties derived from the up-to-date checkpoint conditions. [file 1752-0509-6-129-S1.pdf]

Additional file 1 – Essential ordered properties derived from checkpoints

A state transition is denoted as Gene A: X → Y representing that the value of the gene A is updated from X to Y. Each row refers to a sequential ordered property that a state transition in a state transition #1 column should precede a state transition in a state transition #2 column. All properties of a checkpoint must be satisfied in an ascending order to meet the checkpoint, but more than one sub-properties in a property can be satisfied in any order. For example, the property 1 should hold to check if property 2 holds or not, but two sub-properties in the property 4 (i.e., Clb2 activation should precede Cdc20; Mcm1 activation should precede Cdc20 activation) can be satisfied in any order. Every property is translated into a temporal logic formula to be used as an input to model checking. If model checking detects a state transition that violates a property, such out-of-order transition is called a hazard. If not, a hazard-free model is called timing-robust.

(A) S and G2 phase checkpoint

Property	State transition #1	State transition #2	Reference
1	Cln2: 0 → 1	Sic1: 1 → 0	1-4
2	Sic1: 1 → 0	Clb5: 0 → 1	
3	Clb5: 0 → 1	Clb2: 0 → 1	

(B) M-metaphase checkpoint

Property	State transition #1	State transition #2	Reference
4	Clb2: 0 → 1	Cdc20: 0 → 1	2-8
	Mcm1: 0 → 1	Cdc20: 0 → 1	

(C) M-telophase checkpoint

Property	State transition #1	State transition #2	Reference
5	SBF: 1 → 0	Cln2: 1 → 0	2,5,9-14
	MBF: 1 → 0	Clb5: 1 → 0	
	Cdc20: 0 → 1	Swi5: 0 → 1	
6	Cln2: 1 → 0	Sic1: 0 → 1	
	Clb5: 1 → 0	Sic1: 0 → 1	
	Swi5: 0 → 1	Sic1: 0 → 1	
7	Sic1: 0 → 1	Clb2: 1 → 0	
8	Clb2: 1 → 0	Cdh1: 0 → 1	
	Clb2: 1 → 0	Mcm1: 1 → 0	
9	Cdh1: 0 → 1	Cdc20: 1 → 0	
	Mcm1: 1 → 0	Cdc20: 1 → 0	

References

1. Verma, R., Annan, R., Huddleston, M., Carr, S., Reynard, G., and Deshaies, R.J.: **Phosphorylation of Sic1p by G1 cyclin/Cdk is required for its degradation and entry into S phase.** *Science* 1997, 278, 455-460.
2. Shirayama M, Toth A, Galova M, Nasmyth K: **APC (Cdc20) promotes exit from mitosis by destroying the anaphase inhibitor Pds1 and cyclin Clb5.** *Nature* 1999, 402:203-207.s
3. Zachariae, W., Schwab, M., Nasmyth, K., Seufert, W.: **Control of cyclin ubiquitination by CDK-regulated binding of Hct1 to the anaphase promoting complex.** *Science* 1998, 282, 1721-1724.
4. Jaspersen, S.L., Charles, J.F., and Morgan, D.O.: **Inhibitory phosphorylation of the APC regulator Hct1 is controlled by the kinase Cdc28 and the phosphatase Cdc14.** *Current Biology* 1999, 9, 227-236.

5. Amon, A., Tyers, M., Futcher, B. and Nasmyth, K.: **Mechanisms that help the yeast cell cycle clock tick: G2 cyclins transcriptionally activate G2 cyclins and repress G1 cyclins.** *Cell* 1993, 74:993-1007.
6. Maher, M., Cong, F., Kindelberger, D., Nasmyth, K. and Dalton, S.: **Cell cycle-regulated transcription of the CLB2 gene is dependent on Mcm1 and a ternary complex factor.** *Molecular and Cellular Biology* 1995, 15:3129-3137.
7. Li F, Long T, Lu Y, Ouyang Q, Tang C: **The yeast cell cycle network is robustly designed.** Proceedings of the National Academy of Sciences of the United States of America 2004, 101:4781–4786.
8. Mangla K, Dill DL, Horowitz MA: **Timing Robustness in the Budding and Fission Yeast Cell Cycles.** PLoS ONE 2010, 5:e8906.
9. Knapp D, Bhoite L, Stillman DJ, Nasmyth K.: **The transcription factor Swi5 regulates expression of the cyclin kinase inhibitor p40SIC1.** *Molecular and Cellular Biology* 1996 Oct;16(10):5701–5707.
10. Koch, C. and Nasmyth, K.: **Cell cycle regulated transcription in yeast.** *Current Opinion in Cell Biology* 1994, 6, 451-459
11. Visintin, R., Prinz, S. and Amon, A.: **CDC20 and CDH1: a family of substrate-specific activators of APC-dependent proteolysis.** *Science* 1997, 278:460-463.
12. Prinz S, Hwang ES, Visintin R, Amon A.: **The regulation of Cdc20 proteolysis reveals a role for the APC components Cdc23 and Cdc27 during S phase and early mitosis.** *Current Biology* 1998 Jun 18; 8(13): 750-760.
13. Chen KC, Calzone L, Csikasz-Nagy A, Cross FR, Novak B, Tyson JJ: **Integrative analysis of cell cycle control in budding yeast.** *Molecular Biology of the Cell* 2004, 15:3841–3862.
14. Peter M., and I. Herskowitz.: **Joining the complex: Cyclin-dependent kinase inhibitory proteins and the cell cycle.** *Cell* 1994, 79:181-184.
